# Transfer distortions of acoustic emission signals - power relations between the signal parameters and normalized temporal shapes of avalanches

**DOI:** 10.1038/s41598-025-26238-z

**Published:** 2025-11-07

**Authors:** Asmaa A. Azim, Dezső L. Beke, László Z. Tóth, Lajos Daróczi

**Affiliations:** 1 Department of Solid State Physics, Doctoral School of Physics, University of Debrecen, P.O. Box 2, H-4010 Debrecen, Hungary; 2 Physics Department, Faculty of Science Ain Shams University, Abbassia, Cairo, 11566 Egypt

**Keywords:** Acoustic emission, Crackling noises, Power relations between avalanche parameters, Temporal avalanche shapes, Engineering, Physics

## Abstract

Power law correlation between the rising time, $\:R$, and amplitude,$\:\:A$, of the detected acoustic emission, AE, signals is investigated in the framework of a driven damped harmonic oscillator model. It is shown, in contrast to the previous model calculation, that $\:R\sim{A}^{1-{\varphi}_{AE}}$ holds, similarly to the well-known enigma for acoustic emission; $\:E\sim{A}^{3-{\varphi}_{AE}}\:$as well as $\:S\sim{A}^{2-{\varphi}_{AE}}$, where *E* and *A* are the energy and area, and 3 and 2 are the expected exponents from the mean field theory, MFT. The same value $\:{\varphi}_{AE}=1$ was obtained for all the above exponents and transfer distortions cause mechanism independent changes. For the experimental value $\:{\varphi}_{exp}={\varphi}_{AE}+{\varphi}_{o}$ is fulfilled, where $\:{\varphi}_{o}$ is the exponent in the power relation between the amplitude and the rising time of the source function ($\:{\varphi}_{o}=-0.32$ beyond the MFT and $\:{\varphi}_{o}=0\:$in MFT). The calculated value of $\:{\varphi}_{AE}=1$ is in good agreement with experimental data obtained for different structural changes: $\:{\varphi}_{exp}\:0.8\pm\:0.2$. Universal functions, well scaled together, can be obtained for the temporal avalanche shapes at fixed area normalizing the voltage by $\:A$ and the time by $\:R\sim{A}^{1-{\varphi}_{exp}}$. The first part (around the peak) of it is not sensitive to transfer distortions, while the tail region can be heavily distorted.

**Supplementary Information:**

The online version contains supplementary material available at 10.1038/s41598-025-26238-z.

## Introduction

### The detected voltage signal belonging to crackling noise avalanches

Crackling noises occur in a wide variety of structural changes of materials having jerky character^1,2^, like martensitic transformations^3^, deformation failure of materials and earthquakes^4–7^, magnetization of ferromagnetic materials (Barkhausen noise)^8^ or ferroelectric switching^9^, i.e. during intermittent switching of electric, magnetic or elastic field. It is well-known that different parameters of crackling noise avalanches, such as amplitude, energy, size, or duration, are distributed according to a power law, with various exponent values^1–5^. However, it turned out, that the above exponents are quite robust and do not change considerably for different mechanisms^10,11^ and now the focus is on the average temporal shape of avalanches for fixed duration or size, which in principle can be scaled together for common universal functions^4,12^. Furthermore, as a consequence of the self-similarity of crackling noises^1,2^, well-known power law scaling relations exists between the above parameters of the avalanches and it is still an open question how the exponents of these differ for different mechanisms.

The avalanche temporal shape of crackling noises is characterized by the $\:U\left(t\right)\:$function, which is the detected voltage belonging to the source function, $\:v\left(t\right)$, which can be taken as the local interface velocity (proportional to the time derivative of the local strain change) characteristic for the crackling noise emission. Most frequently acoustic emission, or in ferromagnetic materials, magnetic emission signals (AE and ME, respectively) are detected. For acoustic emission, AE, the event source is the phenomenon, which releases the localized elastic energy in the material (typically by plastic shift of an interface with a certain velocity, $\:v\left(t\right)$, which then propagates as an elastic wave). For magnetic emission, ME, the source signal is the time derivative of the magnetization, proportional to the magnetic domain wall velocity $\:v\left(t\right)$ (or to the velocity of an interface separating two structural modifications, twins or phases, with different magnetization), which results in a voltage signal. In both cases the voltage signal (detected by an appropriate piezoelectric transducer or by a detection coil, correspondingly) is proportional to $\:v\left(t\right)$:

1 $$\:U\left(t\right)\propto\:v\left(t\right)=\frac{d{x}_{s}}{dt}\sim{L}_{o}\frac{d{\varepsilon}_{p}}{dt},$$

where $\:{L}_{o}$ is the length of the sample. In (1) the $\:d{x}_{s}$ shift (or the corresponding plastic strain, $\:d{\varepsilon}_{p}$) belongs to a small irreversible change, when the system is slowly driven by an external field. In addition, in these cases the systems respond to driving through intermittent discrete bursts, or avalanches. It is worth mentioning that, if the voltage $\:U\left(t\right)$ belongs indeed to one avalanche then

2 $$\:S\sim\:{\int}_{t_{s}}^{t_{f}}\left|U\left(t\right)\right|dt=\:{\int}_{o}^{T}\left|U\left(t\right)\right|dt$$

(where the $\:T$ duration time is $\:T={t}_{f}-{t}_{s}$), the so-called avalanche area, $\:S$, should be proportional to the (averaged) interface displacements, $\:{x}_{s}$ (see also e.g^4^). According to the definition of the energy

3 $$\:E=\frac{1}{{R}_{o}}{\int}_{o}^{T}{U}^{2}\left(t\right)dt,$$

where $\:{R}_{o}$ is an arbitrary chosen resistance. Thus, the temporal shape of avalanches of AE signals, i.e. $\:U\left(t\right)$ is an essential function for the interpretation of experimental data. Measurements of AE signals can give information about the distributions of the displacements, $\:{x}_{s}$, shift velocity, $\:v$, and the energy belonging to such displacements: the velocity distributions are directly characterized by the $\:U\left(t\right)$ function, while the displacements and energies have the meaning as values integrated over the duration time. In this communication the most important aspects of the following question are addressed: how the measured parameters of the AE signals are related to the source (to the time derivative of local strain changes) and/or to the distortions, due to the attenuation of the propagating acoustic waves created by the source. The results will be analysed by comparison with acoustic emission experimental data on jerky structural changes in shape memory alloys.

### Transfer function for AE signals

**Fig. 1:**
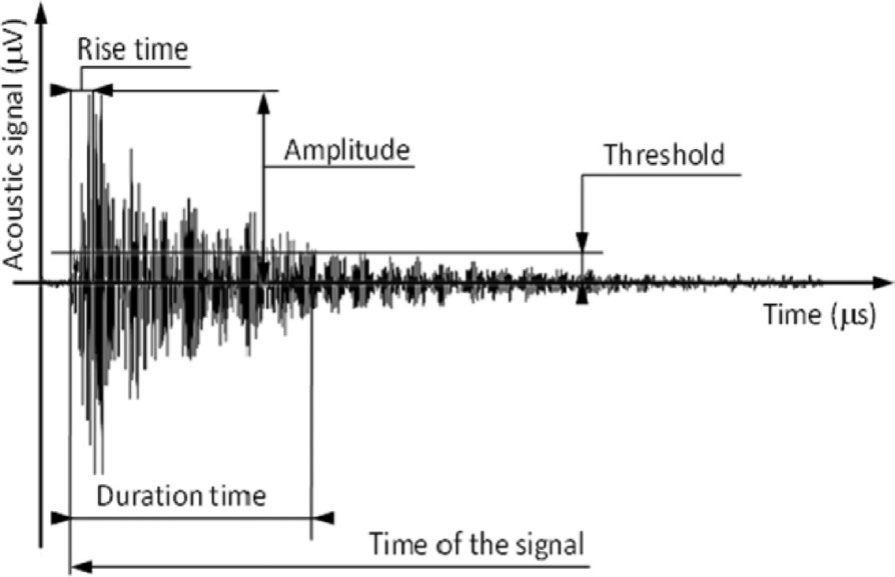
Typical shape of an acoustic emission^3^ signal, schematically.

The measured temporal evolution of the recorded voltage (or its absolute value, $\:\left|U\left(t\right)\right|$) is given as the convolution of the source signal and a transfer function, $\:T\left(t\right)$, describing the effect of the medium + detection system on the wave propagation^13–18^. Then the detected voltage signal, $\:U\left(t\right)$, is given as^14,17,18^:

4 $$\:U\left(t\right)={\int}_{0}^{T}v\left({t}^{{\prime\:}}\right)T\left(t-{t}^{{\prime\:}}\right)dt^{\prime\:},$$

and the integral in general extends over full time (but in our case it should be done for the full duration of the signal, $\:T$). The transfer function, having the form of damped oscillator, can be given as

5 $$\:T\left(t\right)=\text{cos}\left(\omega\:t\right)\text{exp}\left(-\frac{t}{{\tau}_{a}}\right),$$

where $\:{\tau}_{a}$ is the characteristic attenuation time of the acoustic wave and $\:\omega\:$ is a resonant frequency (see e.g. Figure 1 in^14^. The form (5) is suggested by the observed shapes of AE avalanches^5–7^ (oscillating, decaying voltage signals: see Fig. 1), while the shape of the magnetic signals is free of such oscillations, but can be influenced by other distortions of electronic transfer system or by eddy currents^8^. Set-ups for AE measurements have a limited bandwidth and thus might fail to capture the energy (calculated according to (3)) of high frequency events accurately^19,20^. In addition, especially for small signals, the finite threshold values can also distort the detected signal^13,21^. It is a long-standing dilemma; to what extent the measured AE signals reflect the source or the transfer properties, and this is in the focus of our paper.

### The source function of AE signals

In^13^ the authors, on the basis of relations (4) and (5) and using simple model simulations, demonstrated that the power relations between $\:E$ and $\:A$ as well as $\:S$ and $\:A$ (where $\:A$ is the amplitude of the detected signal and $\:E$ denote the experimental values calculated from the detected $\:U\left(t\right)$ function) are given by $\:E\sim{A}^{{\chi}_{E}}$ and $\:S\sim{A}^{{\chi}_{S}}$, respectively. These exponents are different from the values of 3 and 2, predicted by the mean field theory, MFT, (called as enigma for acoustic emission), i.e. $\:{\chi}_{E}=3-{\varphi}_{exp}$ and $\:{\chi}_{S}=2-{\varphi}_{exp}$. In their calculations the following theoretically predicted source function of the average temporal shape of avalanches for fixed area was used^12–14,22^

6 $$\:U\left(t\right)\sim{<v\left(t\right)>}_{S}=atexp\left(-{\left(\frac{t}{{\tau}_{s}}\right)}^{\delta\:}\right),$$

where $\:a$, $\:{\tau}_{s},\:$and $\:\delta\:$ are non—universal (material dependent) constants (which can also be $\:S$*-*dependent^8,22^.$\:\:{\tau}_{s}$ can be interpreted as the characteristic time of the avalanche decay and $\delta{\:=2}$ in mean field approximation^22^. For the time at the maximum, $\:{t}_{m}$, and the maximum value, $\:{v}_{m},\:$of $\:{<v\left(t\right)>}_{S}$ one gets from (6)

7 $$\:{t}_{m}=\frac{{\tau}_{s}}{\sqrt[\delta\:]{\delta\:}}\left(\cong\:\frac{{\tau}_{s}}{\sqrt{2}}\right)\:$$

and

8 $$\:{U}_{m}\sim{v}_{m}=a{t}_{m}\text{exp}\left(-\frac{1}{\delta\:}\right).$$

It can be seen that $\:{t}_{m}$ is proportional to the characteristic time of the avalanche decay and that $\:{U}_{m}$ and $\:{t}_{m}$ are interrelated via the parameters $\:a$ and $\:\delta\:$. In our recent paper^21^, based on relation (8) between $\:{U}_{m}$ and $\:{t}_{m}$ a plausible assumption (self-similar behaviour can be described by power functions^23^, was made, namely $\:\frac{A}{R}\sim{A}^{\varphi\:}$ (where $\:R$ is the experimentally determined rising time) i.e.

9 $$\:R\:\sim{A}^{1-\varphi\:}\sim{A}^{{\chi}_{R}}$$

was supposed. Relation (9) also means that in relation (8) $\:aexp\left(-\frac{1}{\delta\:}\right)$ is a power function of $\:{U}_{m}$: $\:aexp\left(-\frac{1}{\delta\:}\right)$ $\:\sim{U}_{m}^{{\varphi}_{o}}$ (see also below). The assumption (9) was motivated by the search for a one parametrical scaling of (6): according to (9) the voltage as well as the time scales can be divided by $\:A$ as well as by $\:R\sim{A}^{1-\varphi\:}$, respectively.

### Previous results and open questions on power relations

In many publications, using the well-known power law cross-correlation relations (for distortion less cases), valid at least in MFT ($\:S\propto\:{U}_{m}^{2}$ and $\:T\propto\:{U}_{m}$^1,2^ both the voltage and time axes were divided by $\:{S}^{0.5}(\propto\:{U}_{m},\:$as well as $\:\propto\:T$). However, the scaled curves obtained from experimental data on jerky deformation of bulk metallic glasses^4^ or Au and Nb nanocrystals^12^ *did not display universal behaviour*. In contrast to this, knowing the experimentally determined value of $\:{\varphi}_{exp}\:$and using only one scaling parameter from the above two ($\:A$ and $\:R$), very nicely scaled together universal temporal shapes of avalanches belonging to fixed area were obtained in^21,24^. It was true for different AE avalanches observed during martensitic transformation in different shape memory alloys^21^, for plastic deformation both by twinning or by dislocation slip mechanism of Sn^24^ and for simultaneously measured AE and ME avalanches during intermittent motion of single twin boundary in martensitic state of Ni_2_MnGa single crystal^25^. In addition the values of $\:{\varphi}_{exp}$ were the same for all the above AE results ($\:{\varphi}_{exp}\cong\:0.8$*)*, while it was different ($\:{\varphi}_{exp}\cong\:-0.25$) for ME in the same system measured during the same single twin boundary motion between two martensite variants^25^. This difference motivated the authors of^25^ to derive a finite (but different from zero) $\:\varphi\:$ value, $\:{\varphi}_{o}$, for distortion-less cases from the theory beyond the mean field, and $\:{-0.75\le\:\varphi}_{0}\le\:0$ value was obtained ($\:{\varphi}_{o}=0\:$in MFT and beyond MFT $\:{\varphi}_{o}$ changes within the given interval depending on the mechanism of the noise; see also below). In addition, in^21^, using (9) and the definitions of *E* and *S* power relations,

10 $$\:E\propto\:{A}^{3-{\varphi}_{exp}}={A}^{{\chi}_{E}}$$

and

11 $$\:S\propto\:{A}^{2-{\varphi}_{exp}}={A}^{{\chi}_{S}}$$

were deduced. Thus, the so-called energy-amplitude and size-amplitude enigma for acoustic emission, remained open (the values of $\:{\chi}_{E}$ and $\:{\chi}_{S}$ are close to 2 and 1, respectively) suggesting also that the transfer problems are quite different for acoustic and magnetic signals. Nevertheless, the constancy of all the $\:{\varphi}_{exp}$ values for AE during martensitic transformation, plastic deformation or twin boundary motion in different materials, *suggests that distortions due to the transfer effects cause quite similar*,* mechanism independent*,* changes* in the power of cross-correlations between different avalanche parameters.

**Fig. 2:**
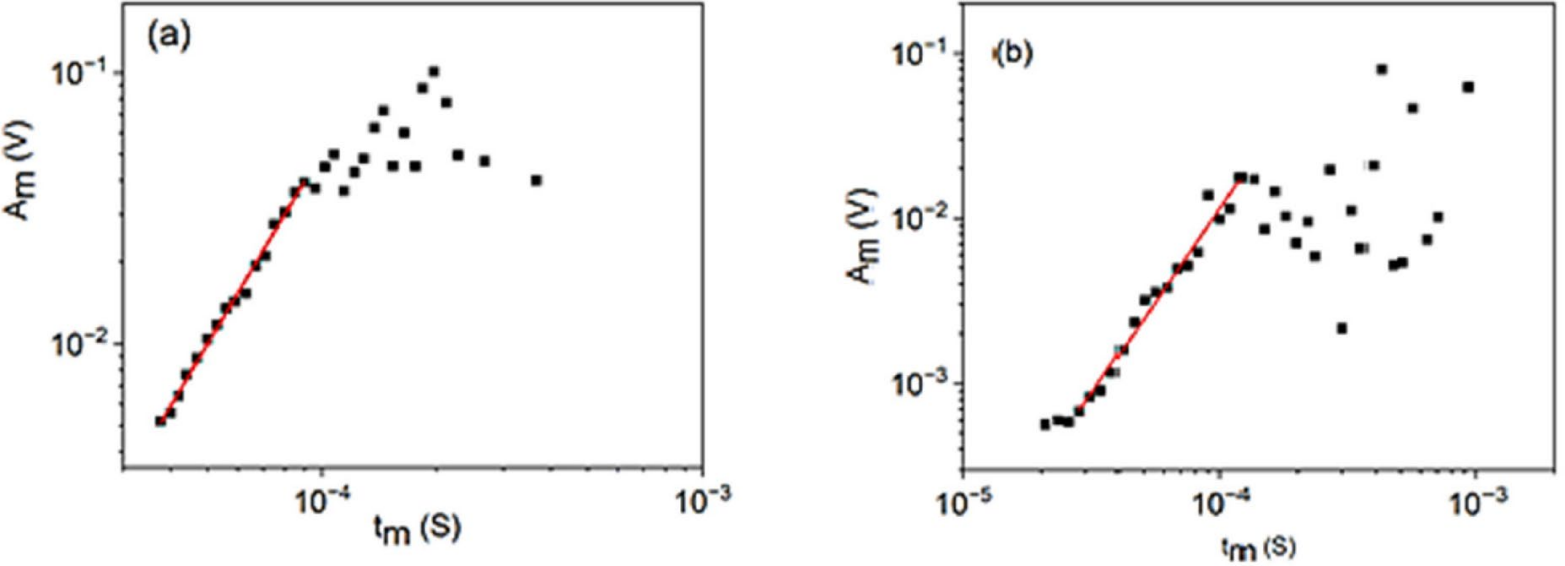
Power relations between the amplitude $\:A(\equiv\:{A}_{m})$ and rising time $\:R\equiv\:\left({t}_{m}\right)$ (on log-log scale) for cooling **(a)** as well as heating **(b)** during martensitic transformations in Ni_45_Co_5_Mn_36.6_In_13.4_ **(a)** as well as Ni_49_Fe_18_Ga_27_Co_6_ (b) shape memory alloys^21^. The fitted straight lines give the average values as $\:{\varphi}_{exp}={\varphi}_{AE}{+\varphi}_{o}=0.7\pm\:0.2.$ (The scatter of points above the fitted regions was interpreted in^21^ by the effect of overlapping subsequent avalanches.).

In this paper, we investigate first the power relations between $\:A$ and $\:R$. Relation (9) was confirmed experimentally in two different alloy systems^21^ with an average value $\:{\varphi}_{exp}=0.7\pm\:0.2$, as it is illustrated in Fig. 2, suggesting that the transfer distortions are similarly important in the relation between $\:A$ and $\:R$^21,25^, as for the exponents in the power relations between $\:E\:$and $\:A$ as well as $\:S$ and $\:A$ as described in^13^. On the other hand, it was concluded from model calculations in^13^ that $\:\varphi\:=0$ in (9) (i.e. this exponent is not influenced by transfer effects). Thus, the first, main question to be answered is: what is the effect of transfer distortions on the power exponent in Eq. (9). In addition, while in^13^ it was concluded that the power law probability density distributions of the energy and size of avalanches were invariant (with the plausible distinction on the duration time distribution), the transfer effects on the averaged temporal shape of avalanches were not investigated. Thus, we will perform such analysis too.

We will start from the theoretically predicted averaged temporal velocity of avalanches of fixed size$\:,\:$as it is given by (6) ($\:{U\left(t\right)\sim<v\left(t\right)>}_{S}=\frac{d{x}_{s}}{dt}\:$) , and put its integral into the equation of a damped harmonic oscillator (having *ω*_*o*_ ringing frequency and *τ*_*a*_ attenuation time: see also Eq. (5)) in a form of $\:{F}_{D}=-D{x}_{s}$ driving force ($\:D$ is the spring constant: see also below). We will use the transfer and source functions as given by Eqs. (5) and (6). This choice is confirmed by detailed simulations in^13^, where the effect of different transfer and source functions were investigated in detail. It was concluded that this choice provides the most important characteristics relevant to the problem addressed here. Furthermore, the use of (5) is also supported by the fact that it describes best the AE signal shapes observed in experiments^5–7^, while the form of (6) is the result of rigorous theoretical description based on elastic-interface model using Functional Renormalization Group theory beyond the MF approximation^22^.

We will investigate the relation between the experimentally determined maximum amplitude and rising time and calculate the values of $\:\varphi\:$ as the function of $\:\frac{{\tau}_{a}}{{\tau}_{s}}$ and $\:{\omega}_{o}$. It will be shown that the AE distortion effects lead to $\:{\chi}_{AE}=1$ in mean field approximation (with $\:{\varphi}_{o}$*=0* and $\:\delta\:=2,\:$which also means that in (8) $\:aexp\left(-\frac{1}{\delta\:}\right)=const.$, i.e. $\:{U}_{m}\sim{t}_{m}$ in distortion-less cases). The experimental values, taking also into account that beyond the mean field theory $\:{\varphi}_{o}\:-0.32$, are expected to be given by $\:{\varphi}_{exp}={\varphi}_{AE}+{\varphi}_{o}$. Thus, the value $\:{\varphi}_{exp}=0.68$ is in excellent agreement with data obtained from number of acoustic emission experiments in different materials ($\:{\varphi}_{exp}=0.8\pm\:0.2$).

## The value of $\:{{\varphi}}_{\varvec{o}}$ beyond the mean field theory

Before the description of our model calculations, we give a derivation for the value of $\:{\varphi}_{o}\:$ beyond the mean field theory. As it was already mentioned in the previous section, in^25^ an expression for $\:{\varphi}_{o}\:$was derived. It was based on a compatibility criterion between the theoretically predicted temporal shapes of avalanches belonging to fixed duration time and area. Here we provide a more simple and straightforward derivation as follows.

It is well-known that the exponents of power cross-correlations between the characteristic parameters of avalanches $\:(A,\:S,\:E)$ can be derived from the self-similarity of avalanches^1,22,26^. Thus, according to^1,22,26^.

12 $$\:T\sim{S}^{\frac{1}{\gamma\:}}, \qquad\:A\sim{T}^{\gamma\:-1},\: \qquad\text{and} \qquad\:E\sim{S}^{2-\frac{1}{\gamma\:}}$$

holds (where $\:T$ is the duration time) and thus

13 $$\:S\sim{A}^{\frac{\gamma\:}{\gamma\:-1}}$$

and

14 $$\:E\sim{A}^{\frac{2\gamma\:-1}{\gamma\:-1}}$$

fulfils. Here $\:\gamma\:$ is a central parameter in the theoretical descriptions of avalanche behaviour and predictions on its value, depending on the type of the basic mechanisms, can change between 1.57 and 2.0^11,22^ ($\:\gamma\:=2\:$belongs to mean field limit). Now, using that the exponent in (13) (or in (14)) can be expressed as $\:{\chi}_{S}=2-{\varphi}_{o}$ (or $\:{\chi}_{E}=3-{\varphi}_{o}$ for undistorted cases), we get

15 $$\:{\varphi}_{o}=\frac{\gamma\:-2}{\gamma\:-1}$$

which is the same as the result derived in^25^. It is clear that for undistorted signals $\:{\varphi}_{o}$ should be negative or zero ($\:{-0.75\le\:\varphi}_{0}\le\:0$). It is zero in MF approximation and according to (8) $\:{v}_{m}{\sim U}_{m}\sim{U}_{m}^{{\varphi}_{o}}{t}_{m}={t}_{m}$ in MF limit. For the comparison with experiments, we have to take the value of $\:\gamma\:$ belonging to the given mechanism. Here, we take $\:\gamma\:=1.76$, as a medium value of $\:\gamma\:$, which corresponds to the model of short range elastic interactions between the moving interface and the pinning points^22^, i.e. $\:{\varphi}_{o}=-0.32$ will be used below.

## Estimation of power relation between $\:{A}\:$and $\:{R}$ from numerical solutions of the driven damped oscillator

For the calculation of the *A* and *R* parameters of the detected signals, we used the simple model of a driven damped harmonic oscillator (see Supplementary Material):

16 $$\:m\ddot{x}=-D(x+{x}_{s})-k\dot{x}.$$

In (16) the $\:-D{x}_{s}$ “driving force” term arises from the acoustic emission source. Since (6) gives the average local stain rate (interface velocity in a model of moving interface in an elastic medium with pinning points), $\:{x}_{s}\:$should be the integral of (6). Furthermore, $\:{x}_{s}\left(t\right)$ is characterized by two input parameters $\:{v}_{m}$ and $\:{t}_{m}\left(=\frac{{\tau}_{s}}{\sqrt{2}}\right)$. In addition, since they are interrelated with $\:{\varphi}_{o}=0$ in MFT (i.e. $\:{v}_{m}\sim{U}_{m}^{{\varphi}_{o}}{t}_{m}={t}_{m}$), what we used here, only one of them was independent. Searching for the solutions of (16), we considered the so-called underdamped solutions (see Supplementary Material).

Simple analytical overdamped solutions of (16) exist only for constant or sinusoidal driving force. Thus, we needed numerical solutions, for which the well-known Euler method was used (see Supplementary Material). The amplitude, $\:A$, and the corresponding time, $\:R$, from the numerical solutions as the function of the parameter $\:\frac{{\tau}_{a}}{{\tau}_{s}}\left(=\frac{{\tau}_{a}}{{t}_{m}\sqrt{2}}\right)$ for fixed values of $\:{\tau}_{a}$ and $\:{\omega}_{o}$ were calculated. The obtained solution, $\:x\left(t\right),$ as the output, will create a voltage signal in the AE sensor, which is proportional to $\:\dot{x}\left(t\right)=\frac{dx\left(t\right)}{dt}.\:$Thus, the amplitude and the corresponding time of the detected AE signal, denoted by $\:A$ and $\:R$ (rising time) belongs to the maximum of $\:\dot{x}\left(t\right)$.

**Fig. 3:**
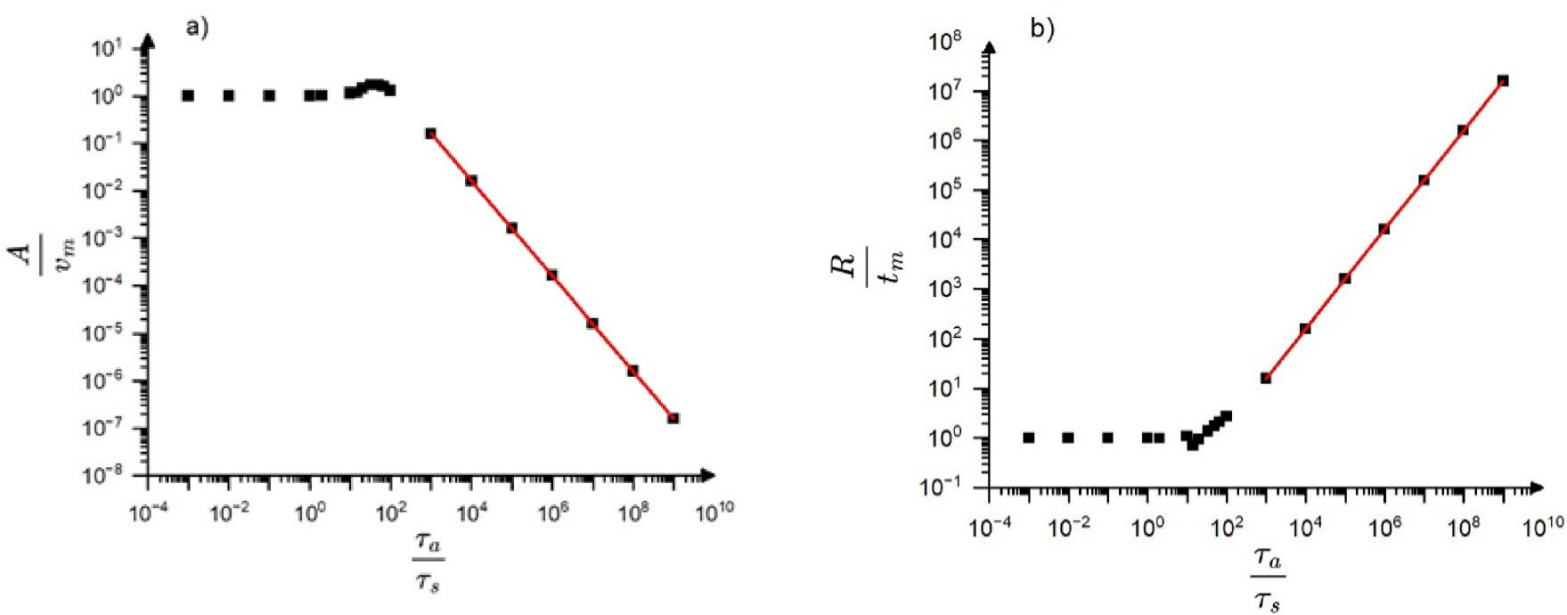
$\:\frac{A}{{v}_{m}}$ as well as $\:\frac{R}{{t}_{m}}$ versus $\:\frac{{\tau}_{a}}{{t}_{s}}\left(=\frac{{\tau}_{a}}{{t}_{m}\sqrt{2}}\right)$ on log-log scale: **(a)** and **(b)**, respectively for $\:{\omega}_{o}={10}^{6}Hz$, $\:{\tau}_{a}=70\mu s$.

Figures 3 shows the $\:\frac{A}{{v}_{m}}$ as well as $\:\frac{R}{{t}_{m}}$ ratios as the function of $\:\frac{{\tau}_{a}}{{\tau}_{s}}(=\frac{{\tau}_{a}}{{t}_{m}\sqrt{2}})\:$parameter on log-log scale (we assumed that the ratio of the input maximum velocity and the maximum time is unity, i.e. $\:\frac{exp\left(\frac{1}{2}\right)}{{a}_{o}}=1$ and $\:{\varphi}_{o}=0$) for $\:{\omega}_{o}={10}^{6}Hz$, $\:{\tau}_{a}=70\mu s$. It can be seen that the above ratios are equal to unity between $\:\frac{{\tau}_{a}}{{\tau}_{s}}={10}^{-4}$ and $\:\frac{{\tau}_{a}}{{\tau}_{s}}=10$ (where there is no distortion and the output maximum, $\:A$, as well as rising time, *R*, are the same as those for the input). On other hand, the $\:\frac{A}{{v}_{m}}$ as well as $\:\frac{R}{{t}_{m}}$ ratios show definite dependence in the range between $\:\frac{{\tau}_{a}}{{\tau}_{s}}=10\:$ and 10^9^: they linearly decrease/increase on the log-log scale with $\:\frac{{\tau}_{a}}{{\tau}_{s}}$. It can be shown that the change of the parameters $\:{\omega}_{o}$ and $\:{\tau}_{a}$ had only minor effect on the behaviour illustrated in Fig. 3 (see Fig. S1 in Supplementary Material, where $\:{\omega}_{o}={10}^{4}Hz$ and $\:{\tau}_{a}=7ms$ was used).

**Fig. 4:**
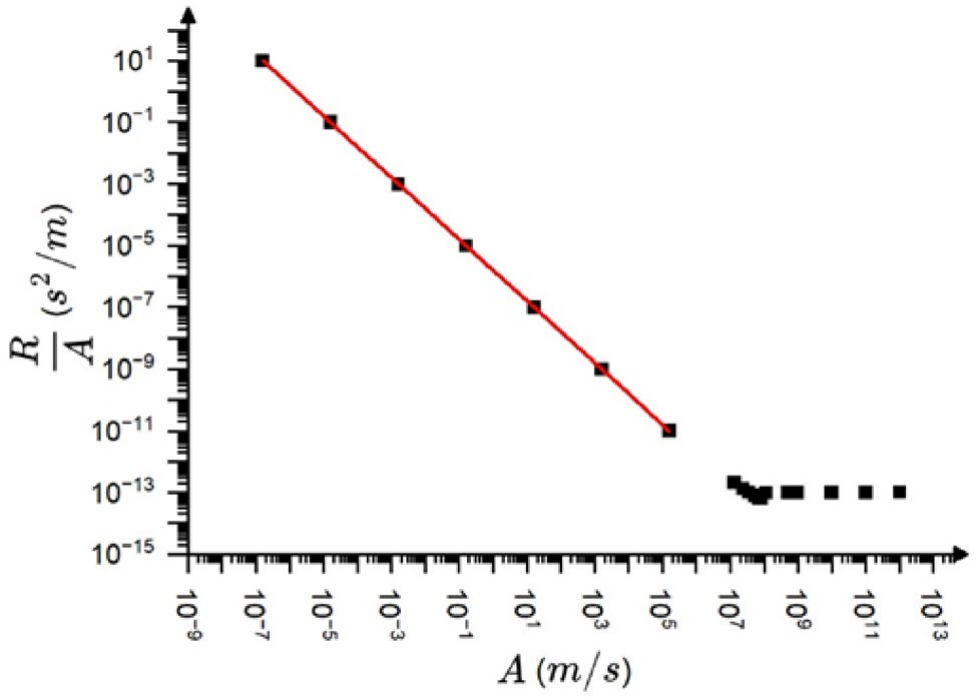
$\:\frac{R\:}{A}$ versus $\:A$ on log-log scale ($\:{\omega}_{o}={10}^{6}Hz$,$\:{\tau}_{a}=70\mu s$). The slope of the fitted line is unity.

Since according to (9) the $\:\frac{R}{A}\sim{A}^{-{\varphi}_{exp}}={A}^{-{\varphi}_{AE}}$ (now $\:{\varphi}_{o}=0$) the slope of the $\:log\:\frac{R}{A}$ versus $\:logA$ plot provides directly the value of $\:{\varphi}_{AE}=1$. Figure 4 shows this function and it can be seen that it is a linear function for small amplitudes (distorted region) with a slope of $\:-1$ in a very wide range of amplitude. Thus, the numerical results provide that $\:{\varphi}_{AE}=1.$ Taking into account this, we can express the $\:{\chi}_{R}$, $\:{\chi}_{S}$ and $\:{\chi}_{E}$ exponents in the following general forms

17 $$\:{\chi}_{R}=1-{\varphi}_{exp}=1-{(\varphi}_{AE}+{\varphi}_{o}),$$

18 $$\:{\chi}_{S}=2-{\varphi}_{exp}=2-{(\varphi}_{AE}+{\varphi}_{o}),$$

and

19 $$\:{\chi}_{E}=3-{\varphi}_{exp}=3-{(\varphi}_{AE}+{\varphi}_{o}),$$

which give e.g. $\:{\chi}_{E}=2-{\varphi}_{o}$ and $\:{\chi}_{S}=1-{\varphi}_{o}$ $\:{(\varphi}_{AE}=1$ independently of the mechanism) for distorted cases, as well as $\:{\chi}_{E}=3-{\varphi}_{o}$ and $\:{\chi}_{S}=2-{\varphi}_{o}$ for undistorted limit.

**Fig. 5:**
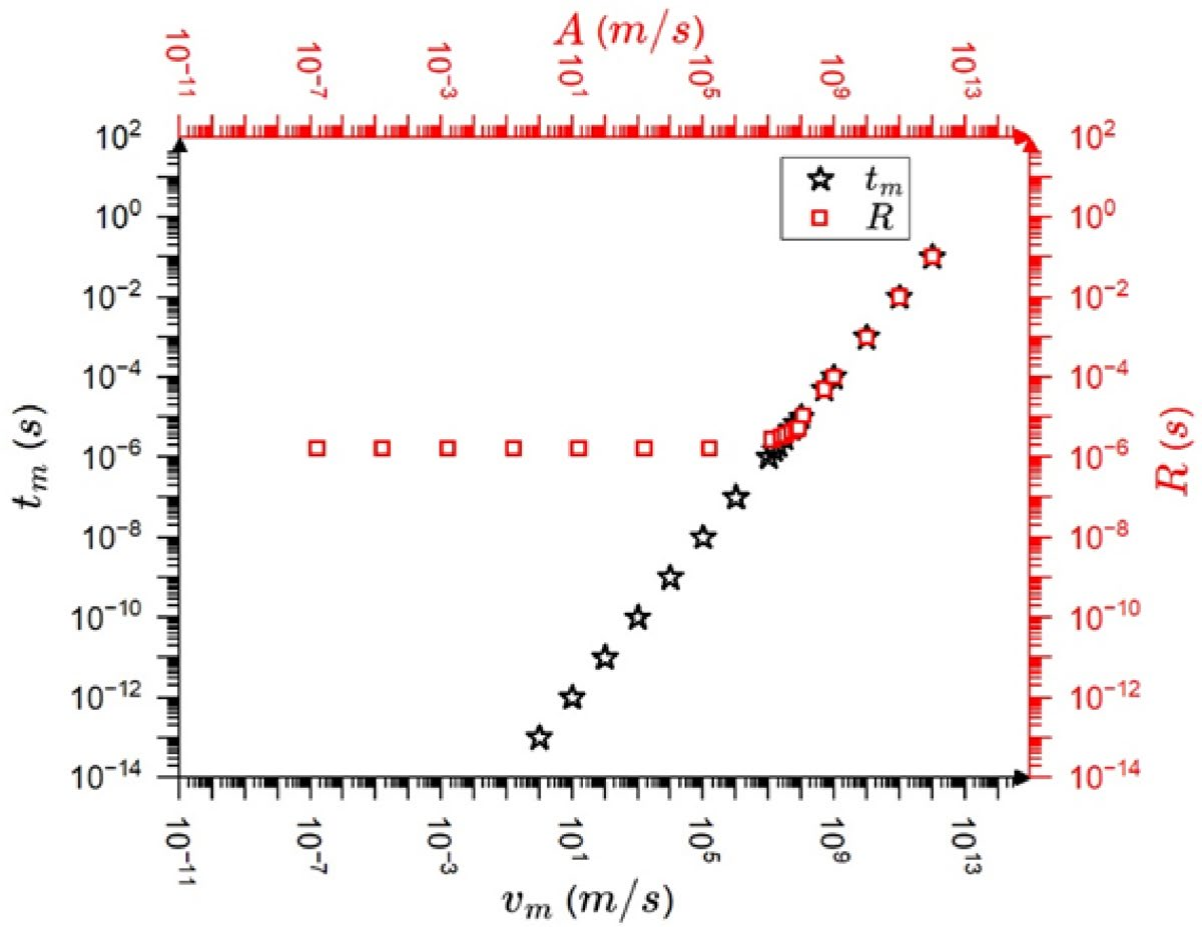
$\:LogR\:$versus $\:logA$ (output: red symbols) as well as $\:\text{log}{t}_{m}$ versus $\:\text{log}{v}_{m}$ (input: black symbols) for $\:{\omega}_{o}={10}^{6}Hz$,$\:{\tau}_{a}=70\mu s$.

In accordance with (18) and (19), $\:{\varphi}_{exp}={\varphi}_{AE}+{\varphi}_{o}=1-0.32=0.68$, which is in very good agreement with the average experimental value $\:0.8\pm\:0.2$, mentioned above. The $\:{\varphi}_{AE}=1$ result is also in agreement with the calculations of^13^, where for the exponents of $\:logE\:$versus $\:logA$ as well as $\:logS$ versus $\:logA$, $\:2$ as well as $\:1$ were obtained for $\:\frac{{\tau}_{a}}{{t}_{m}}\gg\:1,$ respectively. On the other hand, the exponent of the $\:logA$ versus $\:logR\:$was the same as the one for the input (i.e. for the source function) and was equal to unity (i.e. $\:{\varphi}_{AE}=0)$ in^13^ (see Fig. 3f in^13^. In order to illustrate that this is in contrast to our results see Fig. 5, which is equivalent to Fig. 3f of^13^. It can be seen that the $\:logR$ versus $\:logA$ function agrees well with the $\:\text{log}{t}_{m}$versus $\:\text{log}{v}_{m}$ plot for large values of $\:A$ with a slope of unity: only this region was fitted in^13^. On the other hand, the another part (which corresponds to small values of $\:A$ where the distortion effects are remarkable) the slope is zero, giving that $\:1-{\varphi}_{AE}=0$, i.e. $\:{\varphi}_{AE}=1.$ This part, although it was indicated in Fig. 3f of^13^, surprisingly was neglected there, and it was concluded that the rise time is unaffected by transfer distortions. In contrast to this on the plot *logS* versus *logA* in Figs. 3d of^13^ this amplitude region was also fitted. It was concluded in^13^ that the slope of the fitted line in this region differs by 1 (according to the enigma) from the values predicted from the MFT and provided also by the fit in the not distorted region. Thus, we can conclude that the main difference between our conclusion and that of^13^ is that the distorted part was also fitted here, like in the S versus A plot or E versus A plots in^13^.

**Fig. 6:**
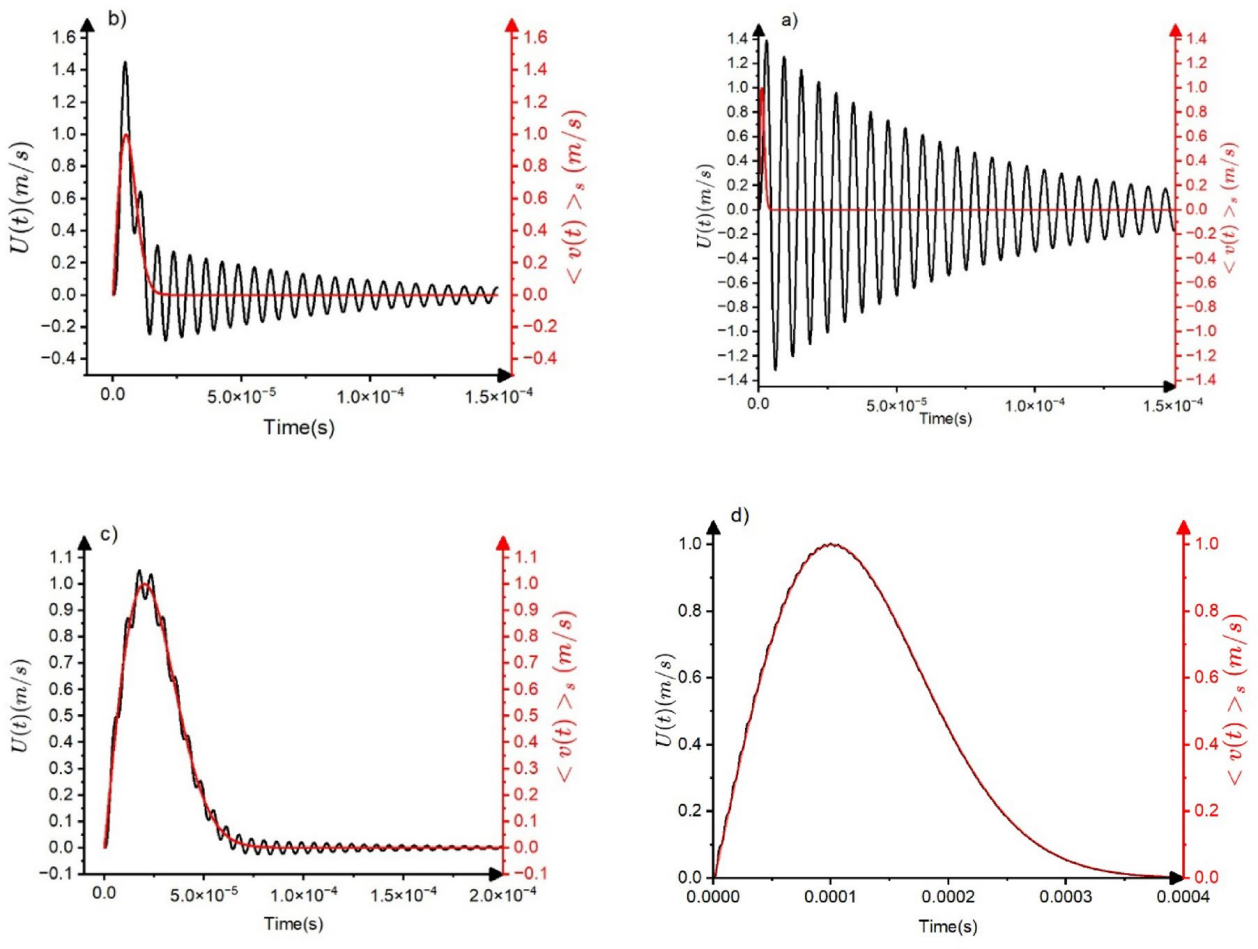
The calculated output signals $\:\dot{x}\left(t\right)\sim U\left(t\right)$ (black curves) ($\:{\omega}_{o}={10}^{6}Hz$,$\:{\tau}_{a}=70\mu s$) for $\:\frac{{\tau}_{a}}{{\tau}_{s}}=45\:$**(a)**;$\:\:\frac{{\tau}_{a}}{{\tau}_{s}}=20\:$**(b)**; $\:\frac{{\tau}_{a}}{{\tau}_{s}}=5\:$**(c)** and $\:\frac{{\tau}_{a}}{{\tau}_{s}}=1$ **(d)**. The input signals are shown by red.

Figure 6a, b, c and d) show, as an illustration, the calculated output signal ($\:\dot{x}\left(t\right)$ as well as the input signal (the $\:{<v\left(t\right)>}_{S}\:$function) for four values of the parameter $\:\frac{{\tau}_{a}}{{t}_{m}\sqrt{2}}=\frac{{\tau}_{a}}{{\tau}_{s}}$. It can be seen that, as expected, in case of (a) ($\:\frac{{\tau}_{a}}{{\tau}_{s}}=45)$ the source signal is heavily distorted. In c) and d) practically the source signal is detected, while (b) can be considered as an intermediate case, i.e. the envelope curve of the output signal is very similar input curve (with a bit smaller maximum value). The input signals are shown by red.

## On the same value of $\:{{\varphi}}_{{e}{x}{p}}$ in the cross correlations

Is it was mentioned above, in $\:R\sim{A}^{1-\varphi\:}$ and in the well-known cross correlations $\:E\sim{A}^{3-\varphi\:},\:$ $\:S\sim{A}^{2-\varphi\:}$ (enigma for AE) the same $\:{\varphi}_{exp}$ values were experimentally obtained. Let us start from the following relations^21^

20 $$\:S\sim{U}_{m}{t}_{m}{S}^{*}={U}_{m}{t}_{m}{I}_{s}$$

and

21 $$\:E\sim{{U}_{m}^{2}{t}_{m}{E}^{*}=U}_{m}^{2}{t}_{m}{I}_{E},$$

where

22 $$\:{S}^{*}={I}_{S}={\int}_{o}^{{T}^{*}}{U}^{*}\left({t}^{*}\right)d{t}^{*}=1.65(1-{e}^{-\frac{{T}^{*2}}{2}})$$

and

23 $$\:{{E}^{*}=I}_{E}\sim{\int}_{o}^{{T}^{*}}{U}^{*2}\left({t}^{*}\right)d{t}^{*}=-\frac{1}{2}{T}^{*}exp\left[-{T}^{*2}\right]+\frac{\sqrt{\pi\:}}{4}\text{erf}{T}^{*}$$

with the following form of the normalized (dimensionless) temporal shape of Eq. (6)

24 $$\:{U}^{*}\left({t}^{*}\right)=1.65{t}^{*}{e}^{-{\left(\frac{{t}^{*}}{{\tau}_{s}^{*}}\right)}^{2}}=1.65{t}^{*}{e}^{-\frac{{t}^{*2}}{2}},$$

where $\:{U}^{*}=\frac{U}{{v}_{m}}\sim\frac{U}{{U}_{m}}$, $\:{T}^{*}=\frac{T}{{t}_{m}},$ $\:{\tau}_{s}^{*}=\frac{{\tau}_{s}}{{t}_{m}}=\sqrt{2}$, $\:{t}^{*}=\frac{t}{{t}_{m}}$, for $\:=2$ (in mean field approximation, see also (7)) and $\:1.65=exp\left(\frac{1}{2}\right).$ Now it was shown in^21^ that if $\:{T}^{*}>2.2$ both integrals are constants (within less than 10%, $\:{I}_{s}\cong\:1.65$ and$\:{\:\:I}_{E}\cong\:\frac{\sqrt{\pi\:}}{4}$, respectively). Furthermore, taking also into account that (see Eq. (22) in^21^

25 $$\:{T}^{*}\cong\:\sqrt{2ln\frac{{U}_{m}}{C}},$$

where $\:C\:$is the threshold, $\:{T}^{*}>2.2$ fulfils if $\:\frac{{U}_{m}}{C}>10$, i.e. by fitting the experimental curves keeping this condition (see also^21^. Since the measured (distorted) duration time is always larger than the true value ($\:{D}^{*}\ge\:{T}^{*})$, the values of the above integrals are not influenced by the transfer distortions. Thus, using the constancy of the above integrals, replacing $\:{U}_{m}$ and $\:{t}_{m}$ in (20) and (21) by $\:A$ and $\:R\sim{A}^{1-{\varphi}_{exp}},$ $\:S\sim{A}^{2-{\varphi}_{exp}}$ as well as $\:E\sim{A}^{3-{\varphi}_{exp}}$ are obtained with the same $\:{\varphi}_{exp}={\varphi}_{AE}+{\varphi}_{o}$ value.

**Fig. 7:**
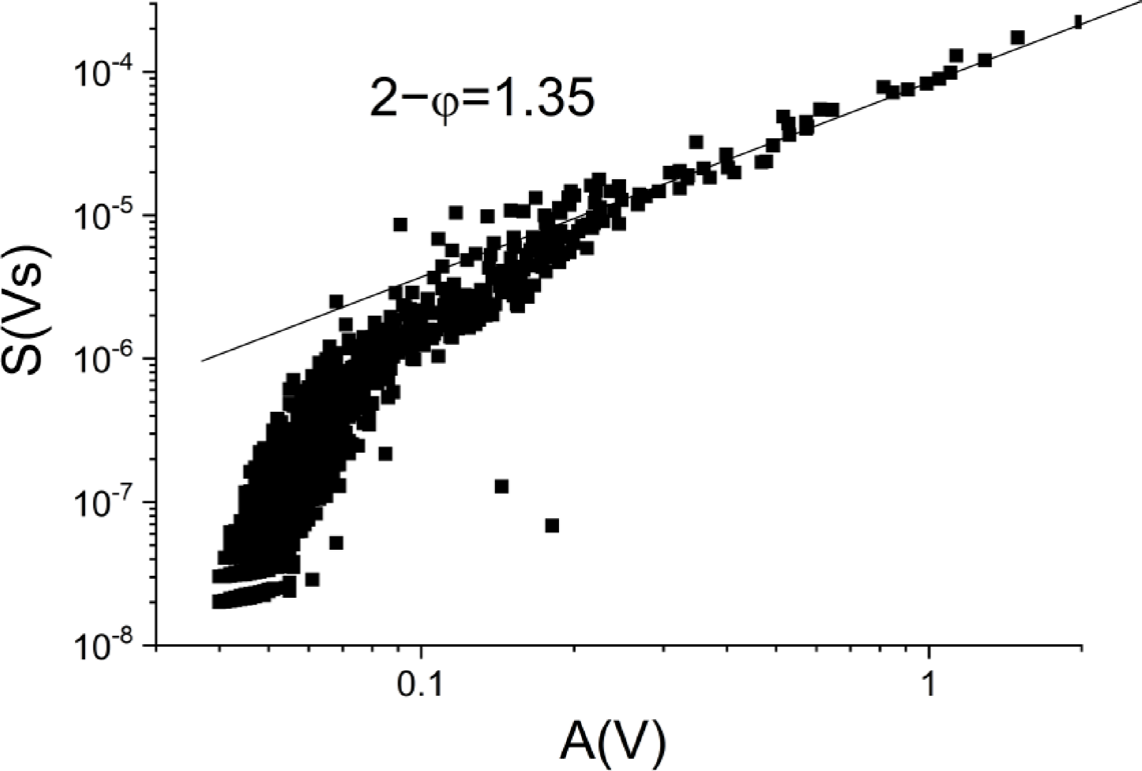
$\:\:LogS$ versus $\:LogA$ from^27^. The insert shows the slope of the straight line fitted, and the downward curvature appearing at small values of *A*. The slope of the fitted straight is 1.35.

For small values of $\:{T}^{*}$ (less than 2.2) the integrals will be smaller than their above limits, which means that on the $\:logS$ versus $\:logA$ or $\:logE$ versus $\:logA$ plots a downward curvature is expected at small values of $\:A\:$(belonging to shorter duration times). Figure 7 illustrates this for AE results obtained during rubber like behaviour of Ni_51_Fe_18_Ga_27_Co_4_ single crystal^27^.

## Distortion effects for the Temporal shapes of avalanches

**Fig. 8:**
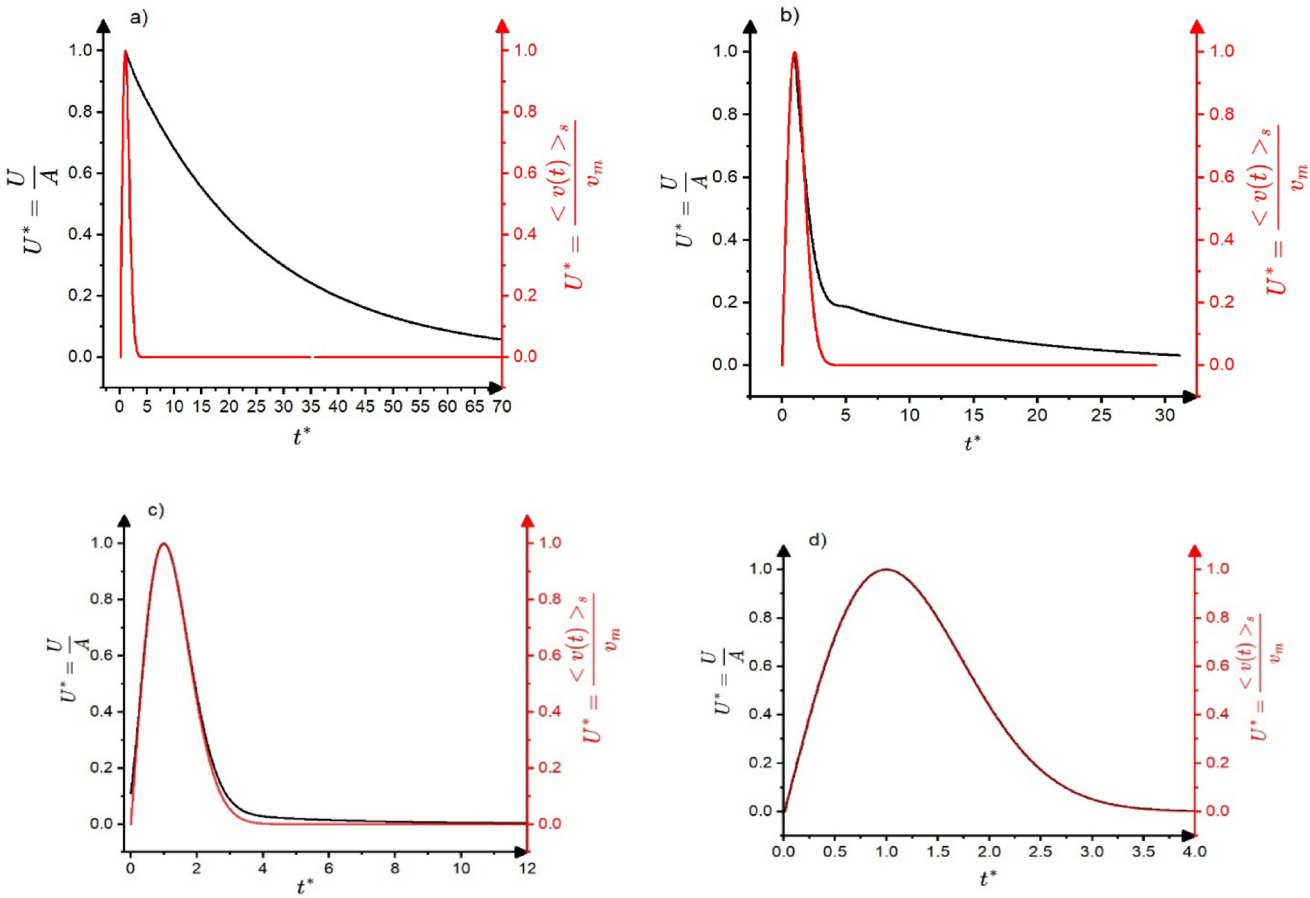
The envelope curves of $\:{U}^{*}=\frac{U}{A}$ versus $\:{t}^{*}=\frac{t}{R}$ (black curves) for the output avalanche shapes at $\:\frac{{\tau}_{a}}{{\tau}_{s}}=45\:$**(a)**;$\:\:\frac{{\tau}_{a}}{{\tau}_{s}}=20\:$**(b)** $\:\frac{{\tau}_{a}}{{\tau}_{s}}=5\:$**(c)** and $\:\frac{{\tau}_{a}}{{\tau}_{s}}=1$ **(d)** For comparison, the input normalized shapes ($\:{U}^{*}=\frac{{<v\left(t\right)>}_{S}}{{v}_{m}}$ versus $\:{t}^{*}=\frac{t}{{t}_{m}}$) are also show with red. At smaller values of $\:\frac{{\tau}_{a}}{{\tau}_{s}}$ ($\:\frac{{\tau}_{a}}{{\tau}_{s}}<20),\:$which corresponds to larger values of input avalanche amplitudes, the first parts of the two curves at around the maxima are very similar, reflecting the undistorted shape even for $\:\frac{{\tau}_{a}}{{\tau}_{s}}=20\:$up to about $\:{t}^{*}=3$.

Based on the above results, shown also in Fig. 6, we can draw conclusions on the transfer distortions of the normalized temporal shapes of avalanches at fixed area. We have to take into account that, according to $\:A\sim{R}^{1-{\varphi}_{AE}}$ in the mean field limit used for the illustration of the transfer effects here, the two experimental scaling parameters, $\:A$ and $\:R$ are linearly related to each other where $\:{\varphi}_{AE}=0$ (see the corresponding parts in Figs. 3,4,5,6, which also correspond to $\:\frac{{v}_{m}}{{t}_{m}}=const.$ i.e. to distortion-less limit). On the other hand, $\:R$ is independent of $\:A$ in the distorted region, where $\:{\varphi}_{AE}=1$ (see the horizontal part of the $\:R$ versus $\:A$ relation in Fig. 5). Thus, normalizing the voltage and time scales by $\:A$ and $\:R$, respectively ($\:{U}^{*}=\frac{U}{A}$, $\:{t}^{*}=\frac{t}{R}$) for large and small values of $\:\frac{{\tau}_{a}}{{\tau}_{s}}$ the behaviour will be different. Of course we are interested in regions where $\:\frac{{\tau}_{a}}{{\tau}_{s}}$ is larger than unity, since for the opposite case the input and output temporal shapes are very similar (see Fig. 6d). Figure 8 shows the normalized input and output shapes for the same three different values of the $\:\frac{{\tau}_{a}}{{\tau}_{s}}$ parameter used in Fig. 6a and 6d. Since in the experiments (taking the averages within certain bins of the area) the oscillatory character is smeared out, in Fig. 8 only the envelope curves are shown for the output shapes. It can be seen that at smaller values of $\:\frac{{\tau}_{a}}{{\tau}_{s}}$, which corresponds to larger values of input avalanche amplitudes since $\:{\tau}_{s}={t}_{m}\sqrt{2}\:$ (see Eq. (7)) and $\:{v}_{m}\sim{t}_{m}$ in accordance with Eq. (10), the first parts of the two curves at around the maxima are very similar, reflecting the undistorted shape even for $\:\frac{{\tau}_{a}}{{\tau}_{s}}=20\:$up to about $\:{t}^{*}=3$. The tail region is more and more governed by the $\:exp\left(-\frac{{t}^{*}}{{\tau}_{a}^{*}}\right)$ (where$\:\:{\tau}_{a}^{*}=\frac{{\tau}_{a}}{{\tau}_{s}}=\frac{{\tau}_{a}}{{t}_{m}\sqrt{2}}$) function, for $\:\frac{{\tau}_{a}}{{\tau}_{s}}>5$.

**Fig. 9:**
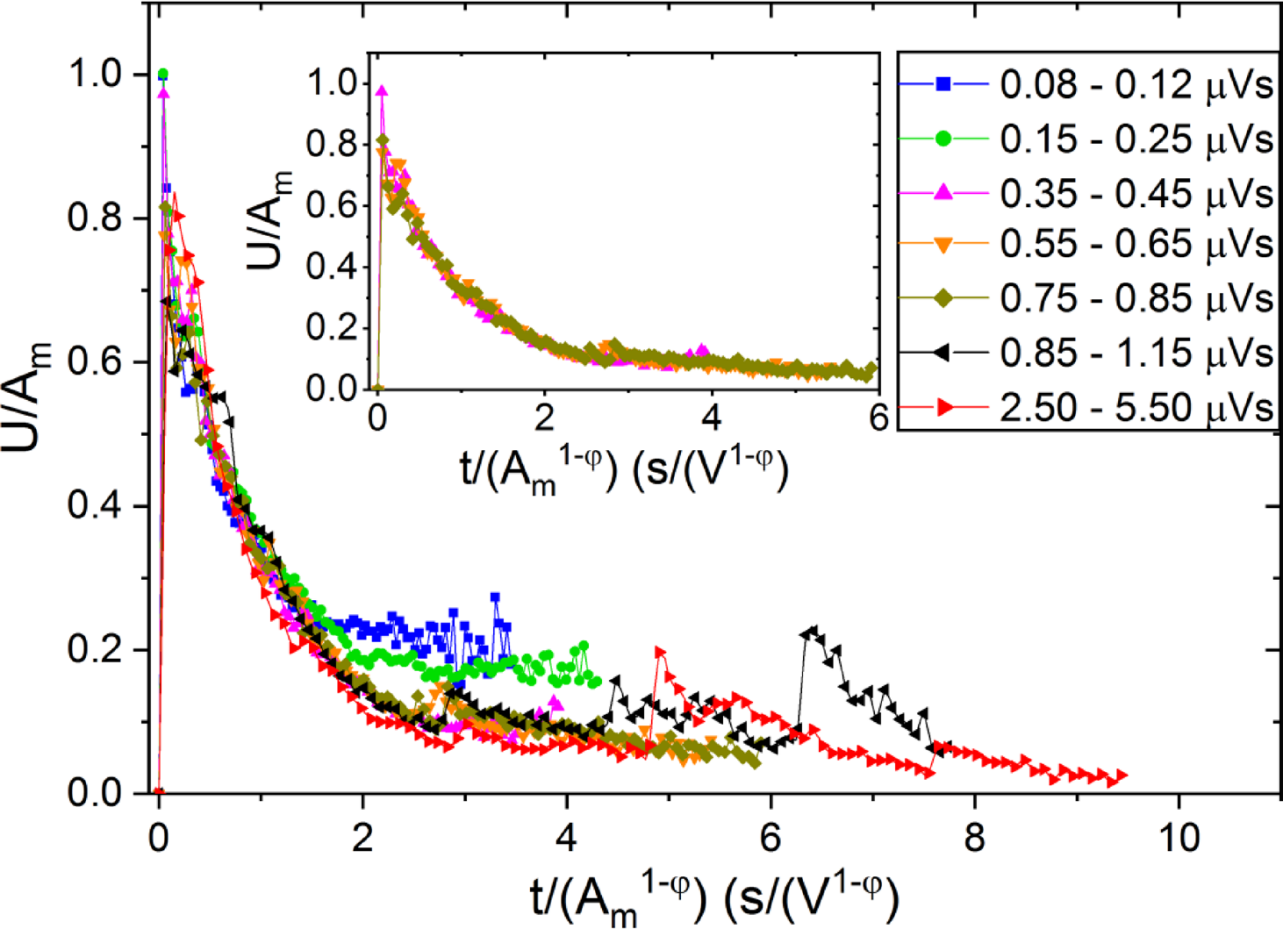
Normalized voltage versus normalized time functions for different bins of avalanche area, $\:S$, obtained from AE measurements during temperature induced martensitic transformation with $\:\varphi\:=0.75$ in Ni_45_Fe_18_Ga_27_Co_6_ shape memory single crystal^21^. The insert shows the same plots without points belonging to the two smallest *S* interval (between 0.08 and 0.12 $\:\mu\:Vs$ as well as between 0.15 and 0.25 $\:\mu\:Vs$). It can be seen that the curves are very well scaled together in the first part (up to about $\:\frac{t}{{A}^{1-\varphi\:}}=1.5\:\frac{s}{{V}^{1-\varphi\:}}$) while they show some fluctuations in tail region for larger values of the reduced time, which can be related to overlapping of sub-avalanches.

The behaviour illustrated in the Fig. 8 was indeed observed in experiments. For comparison with experimental data we have to take also into account that the normalized shapes are expected to be free of threshold effects for area bins in which the median *S* is larger than $\:{S}_{c}\sim{A}_{c}^{2-{\varphi}_{exp}}$, where $\:{A}_{c}>10C$. If this condition fulfils, as it can be seen from Fig. 8, the normalized temporal shapes can be approximately the same around the maxima and deviations (which are dependent on the median value of the area bin) appear in the tail region only. This can be well observed on Fig. 9 (taken from^21^ or on Fig. 1b of^24^. The input signals are shown by red.

Regarding the tail regions (see also Fig. 8), deviations are indeed expected in accordance with Fig. 8b for area bins with median $\:S$ values smaller than $\:{S}_{d}\sim{A}_{d}^{2-{\varphi}_{exp}}$, where $\:{A}_{d}\:$denotes the amplitude below which the $\:\frac{{\tau}_{a}}{{\tau}_{s}}$ is larger than about 10 ($\:{A}_{d}$ can be estimated from $\:\frac{{\tau}_{a}}{{\tau}_{s}}=\frac{{\tau}_{a}}{{t}_{m}\sqrt{2}}=\frac{{\tau}_{a}}{R\sqrt{2}}$, using $\:R\sim A$ for $\:A>{A}_{d}$ according to Fig. 5). In addition, the tail regions can also be influenced by the effect of overlapping of sub-avalanches: the tail of the normalized temporal shapes at fixed area followed Eq. (6) ($\:\sim\text{e}\text{x}\text{p}(-\frac{{t}^{*2}}{2})$, $\:{t}^{*}=\frac{t}{{t}_{m}}\sim\frac{t}{R}\sim\frac{t}{{A}^{1-{\varphi}_{exp}}}$) with exponent 2 for martensitic transformation in Ni2MnGa^25^ or in Ni_49_Fe_18_Ga_27_Co_6_^21,25^. On the other hand, it was obtained in^14^ (from AE signals generated by microcracking events of concrete composites) that the tail could be fitted by $\:\text{e}\text{x}\text{p}(-\frac{t}{{\tau}_{a}})$ type function, characteristic for attenuation of acoustic waves. In addition, as it was also discussed in^14,27^ as an open question, the presence of a slower decay in the tail region can also be related to the contribution of overlapping sub-avalanches, which can lead to Omori-type decay (known from post-seismic slips in earthquakes)^28^, described with a power law: $\:\sim{t}^{-p}$ with $\:p\cong\:1$.

## Dependence of the $\:{\chi}_{{E}}$ and $\:{\chi}_{{S}}$ exponents on the noise mechanism

Since $\:{\varphi}_{o}=\frac{\gamma\:-2}{\gamma\:-1}$ and $\:{\varphi}_{o}$ changes between $\:{-0.75\le\:\varphi}_{0}\le\:0$ corresponding to the mechanism dependent values of $\:\gamma\:$ ($\:1.57\le\:\gamma\:\le\:2$), the $\:{\chi}_{R}$, $\:{\chi}_{S}$ and $\:{\chi}_{E}$ exponents can provide a chance to get conclusion on the mechanisms, independently whether is there any contribution from the transfer distortion or not. Of course, such a conclusion will be stronger for exponents with smaller experimental error. In the previous arguments, we used an average value (averaged over many different systems) as$\:{\:{\varphi}_{exp}=\varphi}_{AE}+{\varphi}_{o}=1+{\varphi}_{o}=0.8\pm\:0.2$, which leads to $\:{\varphi}_{o}=-0.2\pm\:0.2$. However, for a given alloy and for a given mechanism the experimental errors can be considerably less. For instance in^25^ the following values were obtained from the exponents of power relations between $\:E$ and $\:A$, $\:S$ and $\:A$ and $\:R$ and $\:A$: $\:{\varphi}_{o}=-0.25\pm\:0.02$, $\:{\varphi}_{o}=-0.23\pm\:0.03$ as well as $\:{\varphi}_{o}=-0.27\pm\:0.02$, respectively from ME measurements during motion of single twin boundary in Ni_2_MnGa single crystal. In individual AE measurements the $\:{\chi}_{E}$ or $\:{\chi}_{S}\:$values can be determined typically with error bars between $\:\pm\:0.1\:$and $\:\pm\:0.05$^21,25^.

Finally, it is worth mentioning that the values of $\:\gamma\:$ are most frequently calculated from the experimental $\:S\sim{D}^{\gamma\:}\:$relation. It is clear from Fig. 8 (see especially the tails in Fig. 8c) that the condition for the negligible effect on the duration time should be much strict in the above relation than the condition to get reliable temporal shape in the peak area. It also means that the experimental values of the power exponents, $\:{\chi}_{E}$ or $\:{\chi}_{S}$, obtained from AE measurements with $\:{\:\varphi}_{AE}=1$ offer a more accurate estimate for $\:\gamma\:$ using relation (15).

## Conclusions

- the effect of transfer distortions was investigated in a driven damped harmonic oscillator model in MFT limit and it is sown that this alone would result in $\:{\varphi}_{exp}={\varphi}_{AE}=1$ not only in the power exponents between the energy and amplitude ($\:3-{\varphi}_{exp}$) and between the area and amplitude ($\:2-{\varphi}_{exp}$), but between the rising time and amplitude ($\:1-{\varphi}_{exp})$ as well. This means that, in contrast to the conclusions of^13^, the amplitude versus raising time power relation is also distorted, and the transfer distortions cause mechanism independent changes ($\:{\varphi}_{AE}=1$ is the same in all the above cross correlation exponents). Furthermore, the well-known enigma for acoustic emission can be generally stated as “the transfer distortion of the AE signals causes a common, mechanism independent correction in the exponents of power cross-correlations between the detected AE parameters”.
- the transfer distortions appear in the range $\:10<\frac{{\tau}_{a}}{{\tau}_{s}}<{10}^{9},$ where $\:{\tau}_{a}$ and $\:{\tau}_{s}$ denote the attenuation time of the acoustic waves and the decay time of the source function, belonging to fixed area, respectively ($\:{\tau}_{s}={t}_{m}\sqrt[\delta\:]{\delta\:}$, where $\:\delta\:=2$ in MFT and $\:{t}_{m}$ is the time at the maximum of the source function),
- the normalized, dimensionless, temporal shapes of avalanches (obtained by dividing the voltage with the amplitude, $\:A$, and the time with the rising time $\:{R\sim A}^{1-{\varphi}_{exp}}$) for fixed area show that they scale very well together up to about two-three times of the maximum time and are not sensitive to the transfer distortions,
- beyond MF approximation an additional term, $\:{\varphi}_{o}=\frac{\gamma\:-2}{\gamma\:-1}\cong\:-0.32$, appears in the exponents of the power law cross-correlations; $\:{\chi}_{E}=3-{\varphi}_{exp}=3-({\varphi}_{AE}+{\varphi}_{o})$, $\:{\chi}_{S}=2-{\varphi}_{exp}=3-({\varphi}_{AE}+{\varphi}_{o})$ and $\:{\chi}_{R}=1-{\varphi}_{exp}=1-({\varphi}_{AE}+{\varphi}_{o})$, corresponding to $\:E\sim{A}^{{\chi}_{E}}$, $\:S\sim{A}^{{\chi}_{S}}$ and $\:R\sim{A}^{{\chi}_{A}}$ power relations, respectively.

## Supplementary Information

Below is the link to the electronic supplementary material.

Supplementary Material 1

## Data Availability

The datasets generated during and/or analysed during the current study are available from the corresponding author on reasonable request.
