# Supplementary material for: Transfer distortions of acoustic emission signals - power relations between the signal parameters and normalized temporal shapes of avalanches
Source: Sci Rep. 2025 Nov 7;15:39107. doi: 10.1038/s41598-025-26238-z (PMC12594788; doi:10.1038/s41598-025-26238-z)
Supplement: Supplementary file 1 — Supplementary Material 1 [file 41598_2025_26238_MOESM1_ESM.docx]

SUPPLEMENTARY MATERIAL

**Transfer distortions of acoustic emission signals - power relations between the signal parameters and normalized temporal shapes of avalanches**

Asmaa A. Azim^1,2^, Dezső L. Beke^1*^, László Z. Tóth^1^, Lajos Daróczi^1^,

^1^ Department of Solid State Physics, Doctoral School of Physics, University of Debrecen,

P.O. Box 2, H-4010 Debrecen, Hungary,

^2^ Physics Department, Faculty of Science Ain Shams University, Abbassia, Cairo 11566, Egypt.

* Correspondence: [dbeke@science.unideb.hu](mailto:dbeke@science.unideb.hu)

Simple model of a driven damped harmonic oscillator has been used:

$m\ddot{x}=-D(x+x_{s})-k\dot{x}$*,*  (S1)

which can be rewritten into the form

$\ddot{x}+\omega_{o}^{2}(x+x_{s}\left( t \right))+2\beta\dot{x}=0$. (S2)

Here $\omega_{o}^{2}=\frac{D}{m}$ and $2\beta=\frac{k}{m}$ have their usual meaning: $m, D$, and $k$ denote the mass, the spring constant and the damping coefficient, respectively. $\omega_{o}$ is the frequency of the harmonic oscillator for $x_{s}=0$ and $k=0$*,* when the solution of (S2) has the form $x=A_{o} cos\omega_{o}t$. $\beta$ is the so-called attenuation factor (see also below). In (S1) and (S2) the $-Dx_{s}$ “driving force” term arises from the acoustic emission source. Since

$U\left( t \right)\sim{<v\left( t \right)>}_{S}=atexp\left( -\left( \frac{t}{\tau_{s}} \right)^{\delta} \right)$ (S3)

gives the average local stain rate (interface velocity in a model of moving interface in an elastic medium with pinning points), $x_{s}$should be the integral of (S3) (with $\delta=2$ and with the introduction of $v_{m}$ and $t_{m}$ (see in the main text (7) and (8)) i.e.

$x_{s}(t)=\int_{0}^{t} v_{m}exp\left( \frac{1}{2} \right)t^{'}exp\left( -\frac{1}{2}\left( \frac{t^{'}}{t_{m}} \right)^{2} \right)dt^{'}=v_{m}t_{m}exp\left( \frac{1}{2} \right)\left[ 1-exp\left( -\frac{1}{2}\left( \frac{t}{t_{m}} \right)^{2} \right) \right].$ (S4)

It can be seen that $x_{s}\left( t \right)$ is characterized by two input parameters $v_{m}$ and $t_{m}\left( =\frac{\tau_{s}}{\sqrt{2}} \right)$. In addition, since they are interrelated with $\varphi_{o}=0$ in MFT (i.e. $v_{m}\sim U_{m}^{\varphi_{o}}t_{m}=t_{m}$), what we use here, only one of them is an independent parameter. As it is well-known, searching for the solutions of the damped oscillator (for $x_{s}=0$) in the form of $x=A_{o}exp\left( i\omega t \right)$leads to

$\omega^{2}-2\beta\omega i-\omega_{o}^{2}=0$, (S5)

the solution of which is

$\omega_{1,2}=i\beta\pm\sqrt{\omega_{o}^{2}-\beta^{2}=} i\omega_{o}\pm\omega_{o}\sqrt{1-{}^{2}}$, (S6)

where $\omega_{o}=\beta$ and is the damping ratio. Depending on the value of three cases are possible: i) $=1$ (critically damped), ii) $>1$ (overdamped) and iii) $<1$ (underdamped). Since in the first two cases the solutions are exponentially damping function without oscillations (e.g. in case i) the system returns to steady state as quickly as possible with attenuation time, $\tau_{a}=\frac{1}{\beta}=\frac{1}{\omega_{o}}$, i.e. $x=A_{o}exp\left( -\beta t \right)=A_{o}exp\left( -\frac{t}{\tau_{a}} \right)$ ) and the shape of the detected signal contains damped oscillations (see also Fig.1 in the main text), we are interested in solutions of type iii). The general solution of eqn. (S2) (for $x_{s}=0$ and for $<1$) can be given in the form

$x=A_{o}exp\left( -\frac{t}{\tau_{a}} \right)sin\left( {t\omega}_{o}\sqrt{1-{}^{2}}+\varphi\right),$ (S7)

i.e. the frequency is given by $\omega=\omega_{o}\sqrt{1-{}^{2}}$ and the exponential decay is described by*τ_a._*

Simple analytical solutions of (S2) exist only for constant or sinusoidal driving force. Thus, we needed numerical solutions, for which the well-known Euler method [1] was used. The amplitude, $A$*,* and the corresponding time, $R$, from the numerical solutions as the function of the parameter $\frac{\tau_{a}}{\tau_{s}}\left( =\frac{\tau_{a}}{t_{m}\sqrt{2}} \right)$ for fixed values of $\tau_{a}$ and $\omega_{o}$ were calculated. The obtained solution, $x(t),$ as the output, will create a voltage signal in the AE sensor, which is proportional to $\dot{x}(t)=\frac{dx(t)}{dt}\boldsymbol{.}$Thus, the amplitude and the corresponding time of the detected AE signal, denoted by $A$ and $R$ (rising time) will belong to the maximum of $\dot{x}\left( t \right)$**.** The choice of the parameter $\frac{\tau_{a}}{\tau_{s}}$ is supported by the well-known qualitative arguments for the distortions of AE signals [2,3,4,5]: is it expected that these effects are negligible for $\frac{\tau_{a}}{\tau_{s}}\ll1$ and will dominate for $\frac{\tau_{a}}{\tau_{s}}\gg1$. Regarding the experimental values for $\tau_{a}$as well as for $\omega_{o}$ the result of [6] and [7] can be used. In the supplement of [6], from experiments of AE in BaTiO_3_, about $\tau_{a}=20 s,$ as well as in [7], for plastic deformation of Sn, $\tau_{a}=50-90 \mu s$ was published. On the other hand for the $\omega_{o}$ the range 10^4^-10^7^ Hz was given (it depends on the elastic properties of the sample) while from Fig.7 of [7] $\omega_{o}\cong3.5{10}^{6}Hz$ can be taken. In the calculations $\tau_{a}=70 s$ and $\tau_{a}=7ms$ as well as three fixed values of $\omega_{o}$ (${{10}^{5}, 10}^{6}$, and ${10}^{7}Hz$) were used. Figures S1 illustrates that changing the above parameters had only minor effect on the shapes shown in Figure 3 of the main text (calculated for $\omega_{o}={10}^{6}Hz$*,* $\tau_{a}=70s$*)*.


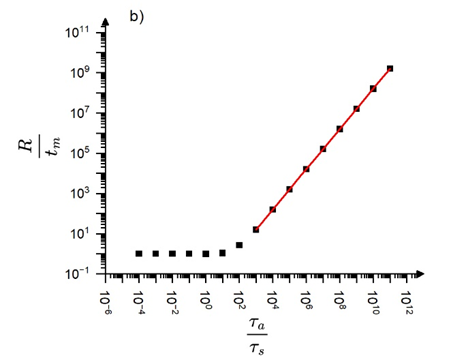

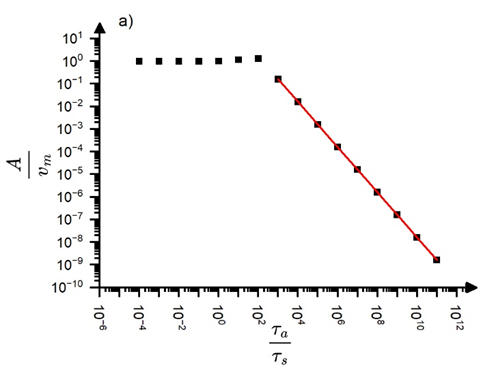


Figure S1. $\frac{A}{v_{m}}$ as well as $\frac{R}{t_{m}}$ versus $\frac{\tau_{a}}{t_{s}}\left( \frac{\tau_{a}}{t_{m}\sqrt{2}} \right)$ on log-log scale ((a) and b)), respectively for $\omega_{o}={10}^{4}Hz$ and $\tau_{a}=7ms.$

References

[1] Butcher John C. (2003). Numerical Methods for Ordinary Differential Equations. New York: John Wiley & Sons. ISBN 978-0-471-96758-3.

[2] C.-C. Vu, J. Weiss, Asymmetric Damage Avalanche Shape in Quasibrittle Materials and Subavalanche (Aftershock) Clusters, *Phys. Rev. Lett.* 125 (2020) 105502. <https://doi.org/10.1103/PhysRevLett.125.105502>

[3] J. Baro, K.A. Dahmen, J. Davidsen, A. Planes, P.O. Castillo, G. F. Natal, E.K.H. Salje, E. Vives, Experimental Evidence of Accelerated Seismic Release without Critical Failure in Acoustic Emissions of Compressed Nanoporous Materials, *Phys. Rev. Lett.* 120 (2018) 245501. [https://doi.org/10.1103/PhysRevLett.120.245501](https://doi.org/10.1103/PhysRevLett.120.245501?_gl=1*saqysp*_ga*MTQwNzIxMzk0OS4xNjc4MjcxNzcz*_ga_ZS5V2B2DR1*MTczOTg3NjQyNi4zNi4xLjE3Mzk4NzY3MzUuMC4wLjE4OTg0NDc2MDc.)

[4] E. Vives, J. Baro, A. Planes, “From labquakes in porous materials to earthquakes*”* in: E.K.H. Salje, A. Setna, A. Planes (Eds.), *Avalanches in Functional Materials and Geophysics*, Springer, 2017, p 31-58, <https://doi.org/10.1007/978-3-319-45612-6_3>

[5] S.M. Kamel, N.M. Samy, L.Z. Tóth, L. Daróczi, D.L. Beke, Denouement of the Energy-Amplitude and Size-Amplitude Enigma for Acoustic-Emission Investigations of Materials, *Materials* 15 (2020) 4556. <https://doi.org/10.3390/ma15134556>

[6] B. Casals, K.A. Dahmen, B. Gou, S. Rooke, E.K.H. Salje, The duration-energy-size enigma for acoustic emission*. Sci. Rep.*  11 (2021) 5590. <https://doi.org/10.1038/s41598-021-84688-7>

[7] L.Z. Tóth, L. Daróczi, T. Y. ElRasasi, D.L. Beke, Clustering Characterization of Acoustic Emission Signals Belonging to Twinning and Dislocation Slip during Plastic Deformation of Polycrystalline Sn, *Materials*, 15 (2022) 6696. <https://doi.org/10.3390/ma15196696>
